# Increased CCL24 and CXCL7 levels in the cerebrospinal fluid of patients with neurosyphilis

**DOI:** 10.1002/jcla.23366

**Published:** 2020-05-17

**Authors:** Xin‐Xin Li, Jing Zhang, Zhao‐Yuan Wang, Si‐Qi Chen, Wei‐Fang Zhou, Ting‐Ting Wang, Xiao‐Yong Man, Min Zheng

**Affiliations:** ^1^ Department of Dermatology Second Affiliated Hospital Zhejiang University School of Medicine Hangzhou Zhejiang China

**Keywords:** CCL24, Cerebrospinal fluid, Chemokine, CXCL7, Neurosyphilis

## Abstract

**Background:**

Monocytes are recruited into the cerebrospinal fluid (CSF) of patients with neurosyphilis, suggesting abnormal chemokine expression. We aimed to investigate the aberrant expression of chemokines in the CSF of these patients.

**Methods:**

CSF and serum samples were collected from patients with neurosyphilis between July 2017 and June 2019 in the Dermatology Department, Second Affiliated Hospital of Zhejiang University. Differences in the expression of 38 chemokines between patients with and without neurosyphilis were detected using RayBio^®^ Human Chemokine Antibody Array C1. CCL24 and CXCL7 levels in the patients’ CSF and serum were further measured using RayBio^®^ CCL24 and CXCL7 ELISA kits.

**Results:**

Ninety‐three CSF and serum samples of patients with syphilis were collected. Antibody array analysis showed that the CSF levels of CCL24 (*P* = .0185), CXCL7 (*P* < .0001), CXCL13 (*P* < .0001), CXCL10 (*P* < .0001), and CXCL8 (*P* < .0001) were significantly higher in patients with than without neurosyphilis. ELISA confirmed significantly higher CCL24 and CXCL7 levels in the CSF of patients with than without neurosyphilis (CCL24: 6.082 ± 1.137 pg/mL vs 1.773 ± 0.4565 pg/mL, *P* = .0037; CXCL7: 664.3 ± 73.19 pg/mL vs 431.1 ± 90.54 pg/mL, *P* = .0118). Increased CCL24 and CXCL7 expression was seen throughout all neurosyphilis stages, had moderate diagnostic efficiency for neurosyphilis, and correlated poorly with CSF cell count and Venereal Disease Research Laboratory titer. CSF CCL24 levels also correlated poorly with CSF protein concentration.

**Conclusion:**

Abnormally high CSF chemokines levels may play a role in the pathogenesis of neurosyphilis.

## 1 INTRODUCTION

Syphilis is a sexually transmitted, chronic infection caused by *Treponema pallidum*, and is characterized by various clinical features involving multiple organs, including the nervous system, which is severely compromised and debilitated.^1^ Neurologic manifestations of syphilis may occur during any stage of infection, and research in animal models has demonstrated that *T pallidum* may disseminate to the central nervous system (CNS) within hours to days after inoculation.^2^

The diagnosis of neurosyphilis is challenging and is primarily based on a combination of clinical and laboratory findings, particularly abnormal CSF parameters. In patients with neurosyphilis, the CSF shows both pleocytosis (mainly lymphocyte accumulation) and mildly increased protein concentration.^3^ The Venereal Disease Research Laboratory (VDRL) assay, performed on CSF, is considered the gold standard for its specificity, but is recognized to have limited sensitivity for the diagnosis of neurosyphilis.^4^ Difficulties in the interpretation of CSF pleocytosis in individuals co‐infected with HIV and syphilis further complicate the evaluation of the relationship between these two diseases. CSF pleocytosis also occurs in individuals with other infections; thus, discerning the cause of pleocytosis in individuals with co‐infections is not always possible.^5^

New potential biomarkers are warranted for discriminating neurosyphilis from other diseases. MicroRNAs (miRNAs) seem to be potential candidate biomarkers, due to their low immunity, good transmission, and ability to cross the blood‐brain barrier. Chen et al reported that miR‐590‐5p, miR‐570‐3p, and miR‐570‐5p are upregulated, while miR‐93‐3p is downregulated in the CSF of patients with neurosyphilis.^6^ Other biomarkers that can assist in the diagnosis of neurosyphilis include chemokines, since the nucleated cells recruited into the CSF of patients with neurosyphilis lead to changes in chemokine expression.^7^ Wang et al previously reported that CXCL13, CXCL10, and CXCL8 levels are elevated in the CSF of patients with neurosyphilis using a quantitative chemokine array and that they could be potential biomarkers for use as complementary diagnostic tools for neurosyphilis.^8^ However, this array did not include other chemokines, such as CCL24, and CXCL7.

In this study, we investigated the abnormal expression of chemokines in the CSF of patients with neurosyphilis in more detail, by using a semi‐quantitative chemokine array that includes overlapping components with that used by Wang et al, but also tests for additional chemokines, such as CCL24 and CXCL7, which have not been previously tested.

## 2 MATERIALS AND METHODS

### 2.1 CSF and serum samples collection

Cerebrospinal fluid and serum samples of patients with syphilis who were referred to the Department of Dermatology, Second Affiliated Hospital of Zhejiang University, School of Medicine, Hangzhou, China, were consecutively collected between July 2017 and June 2019. This study was approved by the Ethics Committee, and written informed consent was obtained from all participants.

The diagnostic criteria of neurosyphilis complied with the Sexually Transmitted Diseases Treatment Guidelines, 2015, of the US Department of Health and Human Services Centers for Disease Control and Prevention (https://stacks.cdc.gov/view/cdc/31403). Non‐neurosyphilis refers to patients with syphilis, including primary, secondary, and tertiary syphilis, who do not meet the diagnostic criteria of neurosyphilis. Different stages of neurosyphilis were subdivided according to the clinical features and associated laboratory test results.^3^ Patients co‐infected with HIV were excluded. Patients with a history of parasitic infections, as well as those with allergic and autoimmune diseases, were also excluded, as CCL24 is a potent eosinophil recruitment chemokine.^9^

### 2.2 Chemokine antibody array

**FIGURE 1:**
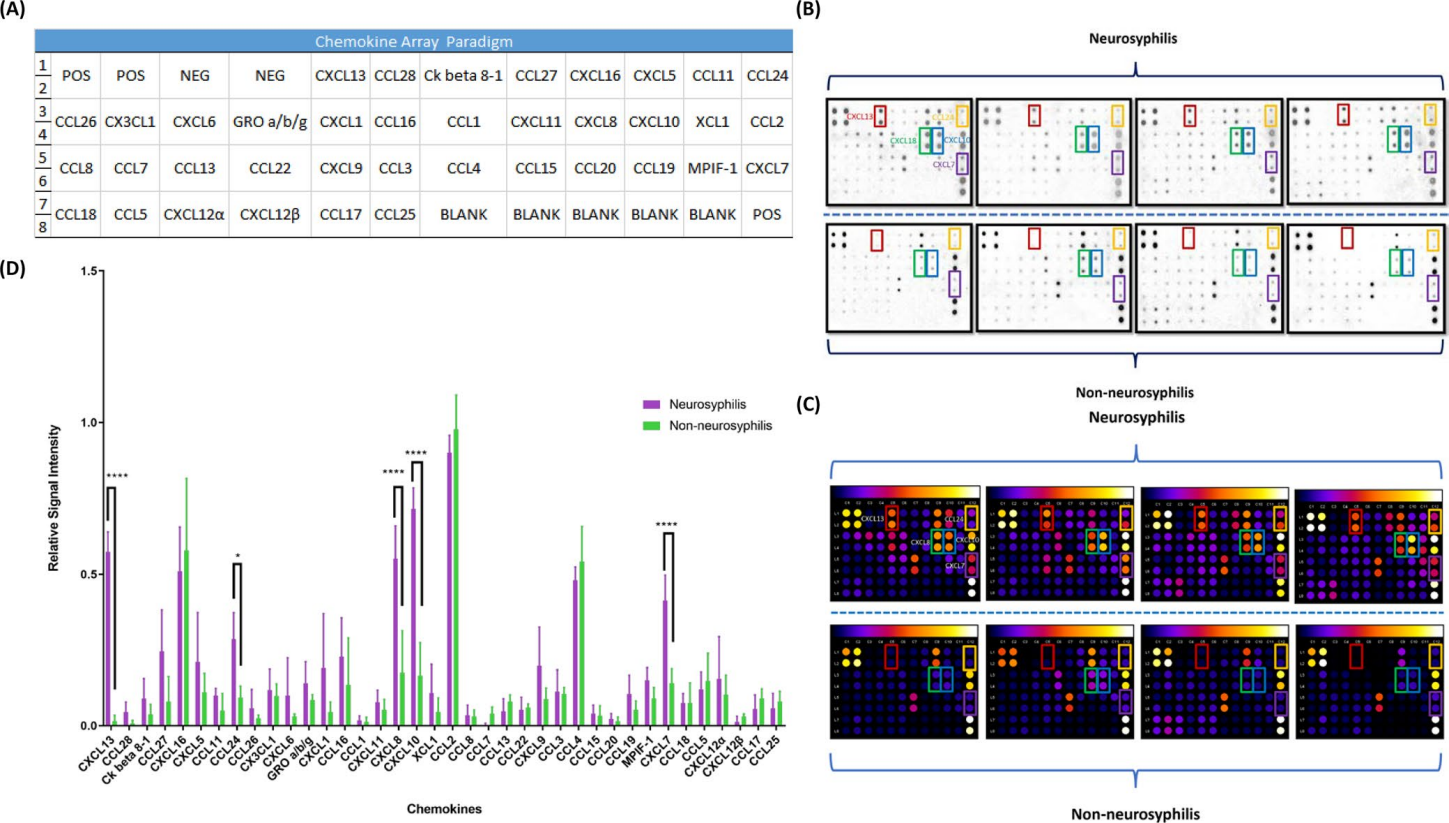
Chemokine array performed on four patients with neurosyphilis and four with non‐neurosyphilis. A, Full chemokine array paradigm. B, Digital images taken with a chemiluminescence imaging system. C, Signal intensities recognized by ImageJ with Protein Array Analyzer plugin. D, Relative signal intensity of 38 chemokines in four patients with neurosyphilis and four with non‐neurosyphilis. CXCL13 is indicated by the red rectangle, CCL24 by the yellow rectangle, CXCL8 by the green rectangle, CXCL10 by the blue rectangle, and CXCL7 by the purple rectangle

The CSF samples were evaluated using a protein array (RayBio^®^ Human Chemokine Antibody Array C1 Kit; RayBiotech), according to the manufacturer's instructions. The full chemokine array paradigm is illustrated in Figure 1A. Digital images of the array (Figure 1B) were taken by means of a chemiluminescence imaging system (ChemiDoc^TM^ MP Imaging system; Bio‐Rad). Signal intensities (Figure 1C) were recognized and analyzed by ImageJ (National Institutes of Health) using the Protein Array Analyzer plugin. The RayBio^®^ ANALYSIS TOOL—Human Chemokine Excel sheet was used to calculate the relative intensities.

### 2.3 Measurement of CCL24 and CXCL7 levels in CSF and serum samples

CCL24 and CXCL7 levels in the CSF and serum samples were quantified by using RayBio^®^ CCL24 and CXCL7 ELISA kits, according to manufacturer's instructions. CSF and serum samples were diluted to meet the detection range.

### 2.4 Statistical analysis

Variations in mean values are expressed as standard errors, and variations in medians are expressed as interquartile range (IQR). Differences between groups were analyzed by Student's *t* test of variance for parametric variables or the Mann‐Whitney *U* test for non‐parametric variables. The capability of biomarkers to diagnose neurosyphilis was evaluated by means of receiver operating characteristic (ROC) curves. Spearman correlation analysis was performed to evaluate the relationship between the levels of CSF chemokines and CSF total protein concentration, cell count, VDRL titer, and serum rapid plasma reagent (RPR) titer. All statistical calculations were performed using GraphPad Prism 7.0 (GraphPad Software), with statistical significance set at *P* < .05.

## 3 RESULTS

### 3.1 CXCL13, CCL24, CXCL8, CXCL10, and CXCL7 levels are selectively elevated in the CSF of patients with neurosyphilis

Cerebrospinal fluid and serum samples from 93 patients with syphilis were available for this study. The characteristics of the overall study population are shown in Table S1, while Table S2 shows a listing of demographic and clinical characteristics of the full study population.

Eight CSF samples from four patients diagnosed with neurosyphilis and four diagnosed with syphilis (non‐neurosyphilis) (Table S3) were selected for analysis by chemokine antibody array. The levels of CCL24 (*P* = .0185), CXCL7 (*P* < .0001), CXCL13 (*P* < .0001), CXCL10 (*P* < .0001), and CXCL8 (*P* < .0001) in the CSF of neurosyphilis patients were higher than those in the CSF of patients without neurosyphilis (Figure 1D).

### 3.2 CCL24 and CXCL7 CSF levels are significantly higher in patients with neurosyphilis than without neurosyphilis

**FIGURE 2:**
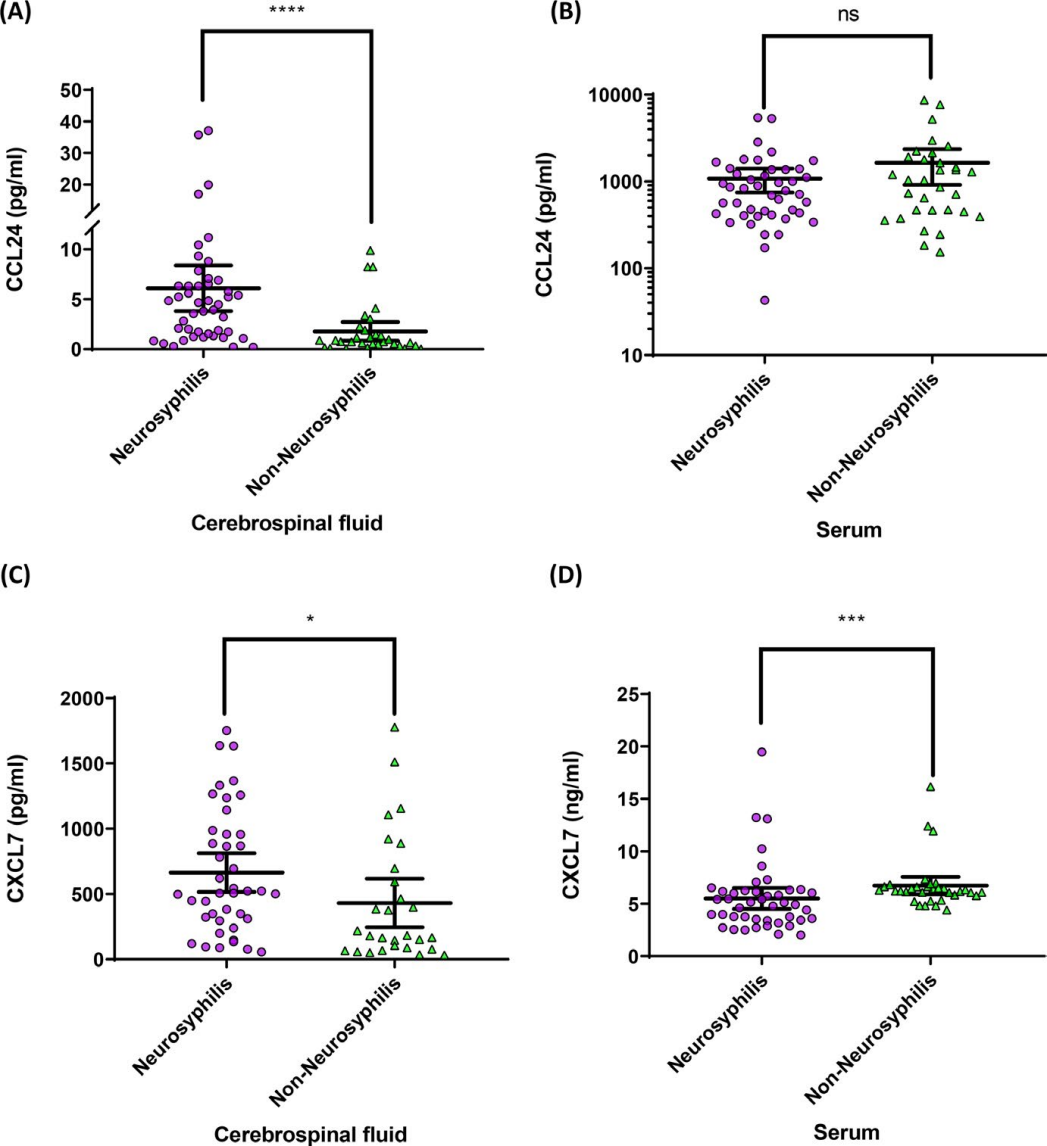
Cerebrospinal fluid (CSF) and serum levels of CCL24 and CXCL7. A and B, CSF (A) and serum (B) levels of CCL24. C and D, CSF (C) and serum (D) levels of CXCL7

We then validated whether the levels of CCL24 and CXCL7 were elevated in the CSF of patients with neurosyphilis, as compared to the levels in those without neurosyphilis, by performing ELISA measurements. The CSF levels of CCL24 and CXCL7 were both statistically significantly higher in patients with neurosyphilis than in those without (CCL24: 6.082 ± 1.137 pg/mL vs 1.773 ± 0.4565 pg/mL, *P* = .0037; Figure 2A; CXCL7: 664.3 ± 73.19 pg/mL vs 431.1 ± 90.54 pg/mL, *P* = .0118; Figure 2C).

We also measured the levels of CCL24 and CXCL7 in the serum of patients. There was no significant difference in serum CCL24 levels between groups (1079 ± 165.2 pg/mL vs 1641 ± 356.2 pg/mL, *P* = .2830; Figure 2B); however, serum CXCL7 levels were lower in patients with than without neurosyphilis (5.511 ± 0.4946 ng/mL vs 6.741 ± 0.4094 ng/mL, *P* = .0004; Figure 2D).

### 3.3 CCL24 and CXCL7 CSF levels are not affected by neurosyphilis stages

**FIGURE 3:**
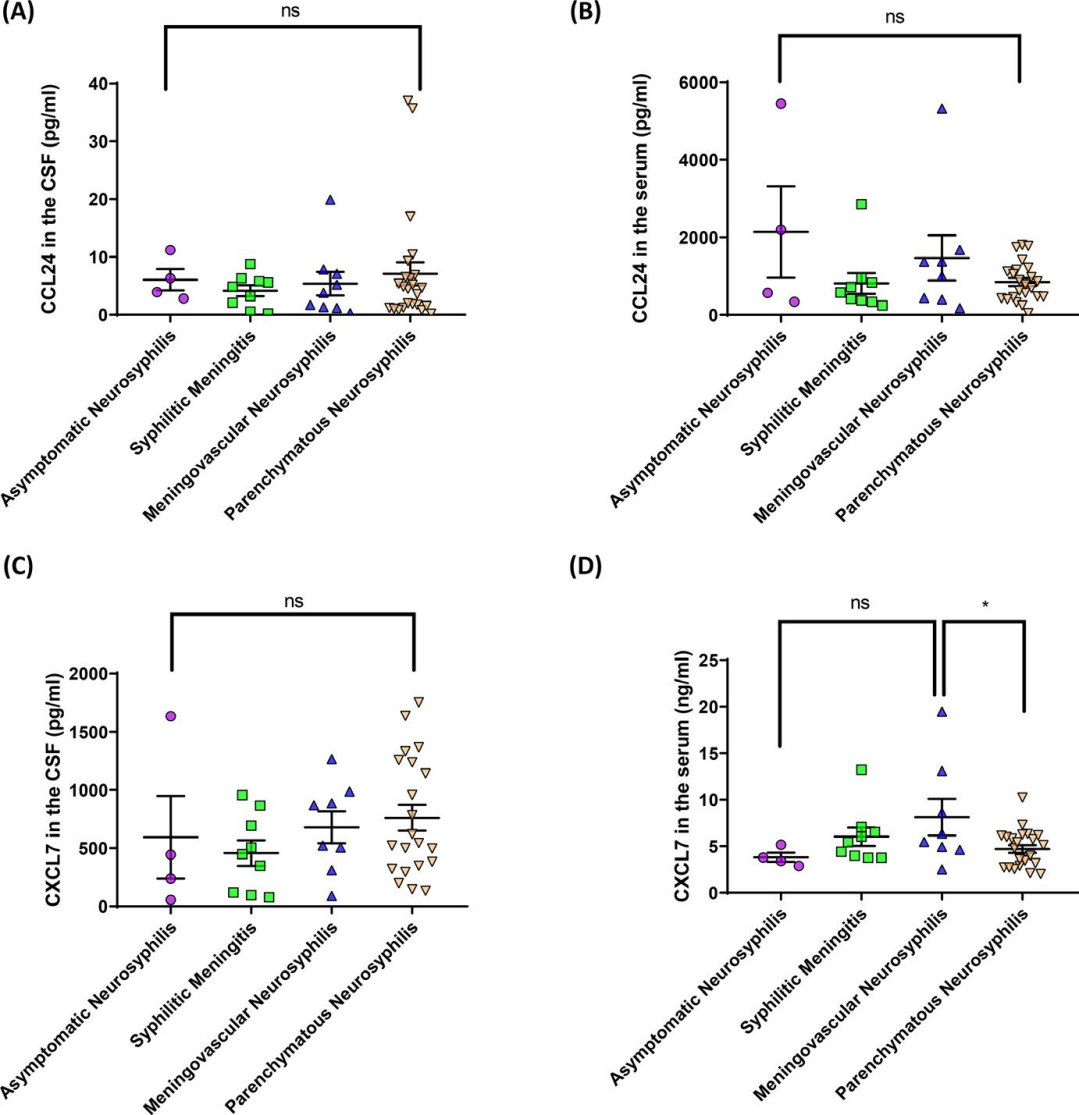
Cerebrospinal fluid (CSF) and serum levels of CCL24 and CXCL7 at different stages of neurosyphilis. A and B, CSF (A) and serum (B) levels of CCL24 at different stages of neurosyphilis. C and D, CSF (C) and serum (D) levels of CXCL7 at different stages of neurosyphilis

The levels of CCL24 and CXCL7 in the CSF and serum of neurosyphilis patients did not differ significantly according to different disease stages (Figure 3A‐C), with the exception of CXCL7 levels in the serum, which differed significantly between meningovascular and parenchymatous neurosyphilis (*P* = .0441; Figure 3D).

### 3.4 Diagnostic value of CCL24 and CXCL7 CSF and serum levels

**FIGURE 4:**
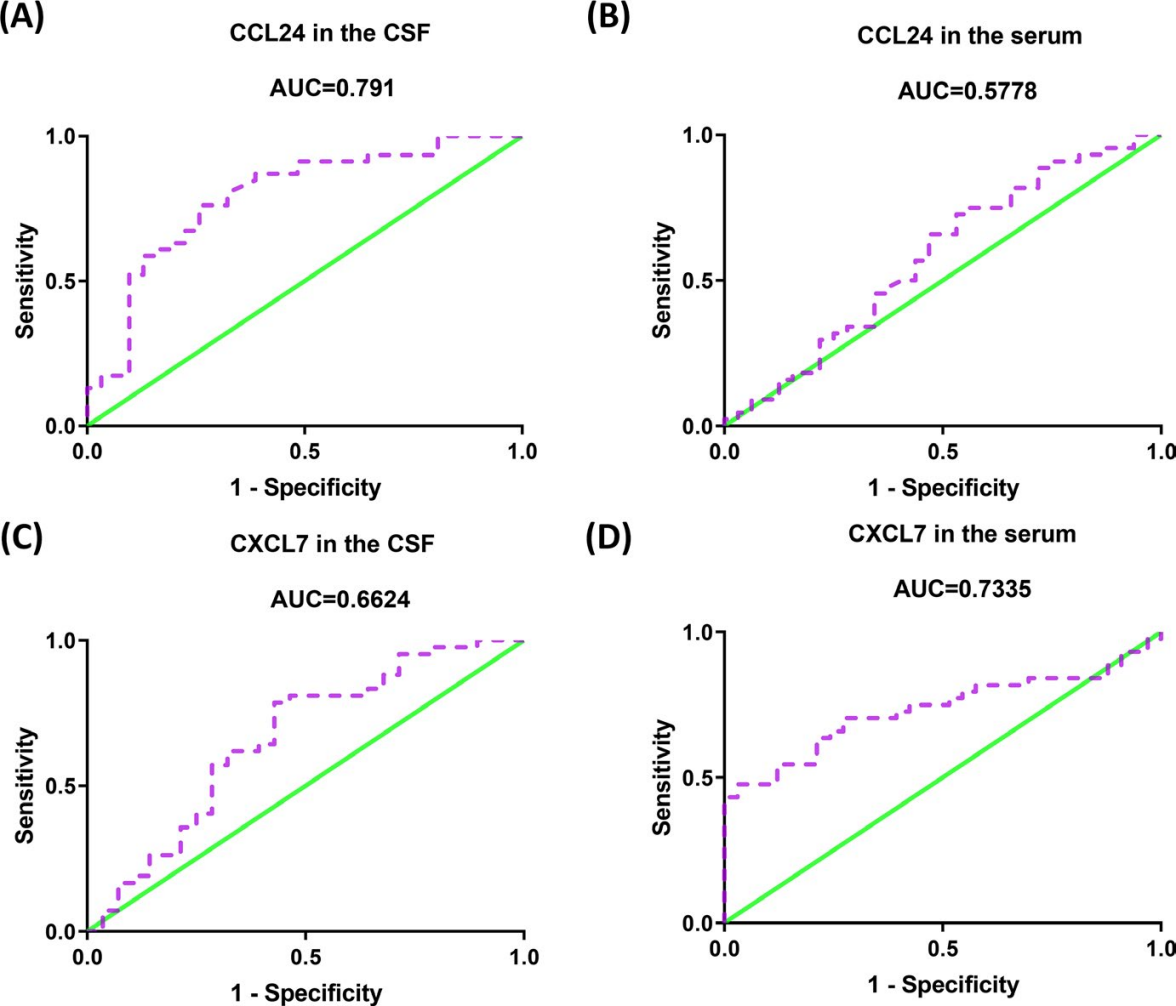
Receiver operating characteristic curve analyses of cerebrospinal fluid (CSF) and serum chemokine biomarkers in discrimination of neurosyphilis. A and B, Area under the curve (AUC) of CSF (A) and serum (B) levels of CCL24. C and D, AUC of CSF (C) and serum (D) levels of CXCL7

Receiver operating characteristic curves to evaluate the diagnostic performance of CSF CCL24 levels for neurosyphilis yielded an area under curve (AUC) of 0.791 (95% confidence interval [CI] 0.6867‐0.8974, *P* < .0001; Figure 4A); the optimal cutoff value was 1.49 pg/mL, with a sensitivity of 76.09% (95% CI 0.6123‐0.8741) and a specificity of 74.19% (95% CI 0.5539‐0.8814). The AUC of CSF CXCL7 levels was 0.6624 (95% CI 0.5267‐0.7981, *P* = .0220; Figure 4C); the optimal cutoff value was 229.36 pg/mL, the sensitivity was 78.57% (95% CI 0.6319‐0.897), and the specificity was 57.14% (95% CI 0.3718‐0.7554). The AUC of serum CCL24 levels was 0.5778 (95% CI 0.4439‐0.7116, *P* = .2493; Figure 4B); the optimal cutoff value was 1182.84 pg/mL, with a sensitivity of 72.73% (95% CI 0.5721‐0.8504) and specificity of 46.88% (95% CI 0.2909‐0.6526). The AUC of serum CXCL7 levels was 0.7335 (95% CI 0.6209‐0.846, *P* = .0005; Figure 4D); the optimal cutoff value was 4.41 ng/mL, while the sensitivity was 43.18% (95% CI 0.2835‐0.5897), and specificity was 100% (95% CI 0.8942‐1).

### 3.5 Correlation analysis of CSF CCL24 and CXCL7 levels and total CSF protein concentration, CSF cell count, CSF VDRL titer, and serum RPR titer

CSF CXCL7 levels were significantly and positively associated with total CSF protein concentration (Figure S1E) but not with other parameters. CSF CCL24 levels did not correlate with CSF total protein concentration, cell count, VDRL titer, or serum RPR titer (Figure S1A‐D,F‐H).

## 4 DISCUSSION

The exact mechanism underlying monocyte infiltration into the CSF of patients with neurosyphilis remains elusive. To gain more insight into this matter, we assessed chemokine levels in the CSF from patients with neurosyphilis and non‐neurosyphilis. We found that the levels of CXCL13, CCL24, CXCL8, CXCL10, and CXCL7 were significantly higher in the CSF of those with than those without neurosyphilis.

Our data are consistent with those of Wang et al^8^; additionally, our study identified that CCL24 and CXCL7 levels are increased in the CSF of patients with neurosyphilis. Moreover, we confirmed these protein array outcomes by ELISAs, indicating that levels of these two chemokines are indeed elevated in the CSF of neurosyphilis, regardless of their serum concentrations. Moreover, the CSF levels of CCL24 and CXCL7 were not different among different stages of neurosyphilis.

Using ROC curve analysis, we showed that CSF and serum CCL24 and CXCL7 levels have moderate diagnostic performance for neurosyphilis, although they are not quite suitable as biomarkers for discriminating neurosyphilis from non‐neurosyphilis, given their sensitivity and specificity values.

CCL24 and CXCL7 levels poorly correlated with CSF protein concentration, cell count, VDRL titer, and serum RPR titer. These findings were expected, because CCL24 and CXCL7 are chemotactic for resting T lymphocytes, eosinophils, and neutrophils, but have no chemotactic activity for activated lymphocytes nor for producing antibodies.^10^ The reason why CXCL7 levels in the CSF were positively associated with total CSF protein concentration was not immediately clear, but it is plausible that activated platelets could secrete various growth factors, such as platelet‐derived growth factor, transforming growth factor β‐1, insulin‐like growth factor‐1, vascular endothelial growth factor, basic fibroblast growth factor, and prostaglandin, which not only disrupt the microenvironment of the CNS, but also increase blood‐brain barrier permeability.^11^

Furthermore, the role of the chemokine CXCL13, also known as B‐cell‐attracting chemokine‐1, which is important for B‐ and T‐cell homing,^12^, ^13^ in Lyme neuroborreliosis, can yield insight into that in neurosyphilis. When spirochetes of *Borrelia burgdorferi*, the causative organism of Lyme disease, invade the CNS, spirochetal lipoproteins induce the release of CXCL13 into the CSF by resident mononuclear cells.^14^, ^15^ CXCL13 contributes to the development of ectopic germinal centers within the CNS. B cells are then recruited to the infectious site by a concentration gradient of CXCL13, and CXCL13 also facilitates B‐cell differentiation into plasma cells, inducing pleocytosis in the CSF of patients with neuroborreliosis.^15^

Similar to neuroborreliosis, neurosyphilis also involves spirochetal infections of the CNS.^16^ Several recent studies have shown that CSF CXCL13 concentration is elevated in both HIV‐positive and HIV‐negative patients with neurosyphilis.^14^, ^17^ Recruitment, activation, and differentiation of B cells, as well as the formation of ectopic germinal centers, have also been observed in the CNS of patients with neurosyphilis, suggesting that CXCL13 overexpression provokes a strong humoral response within the CNS, leading to the destruction of neurological and vascular structures.^18^

CXCL10 is induced by IFN‐γ and binds to its receptor, CXCR3, enhancing the innate antimicrobial defense, and attracting and promoting adhesion of T cells. Moreover, it is a target for treatment of autoimmune diseases, such as multiple sclerosis and rheumatoid arthritis.^19^

CXCL8 interacts with its receptors CXCR1 and CXCR2 and enhances microglial matrix metalloprotease production, leading to the breakdown of the blood‐brain barrier and invasion of inflammatory cells, promoting the inflammatory cascade.^20^ CXCL8 plays crucial roles in the development and metastasis of different cancers.^21^

The levels of CXCL10 and CXCL8 in the CSF have previously been shown to be elevated both in patients with neuroborreliosis and with HIV‐negative neurosyphilis.^16^ Moreover, the levels of CXCL13, CXCL8, and CXCL10 were shown to correlate with CSF protein concentration, and after antibiotic treatment, the concentrations of these chemokines were markedly reduced.^8^ The AUCs of CSF CXCL13, CXCL8, and CXCL10 were all approximately 0.9 in a previous study, indicating that these chemokines are not only potential biomarkers as complementary diagnostic tools for neurosyphilis but may also be useful for monitoring therapeutic effects.^8^ Interestingly, CXCL8, as CXCL7, was reported to reduce neutrophil adhesion and migration during inflammation, and thus plays a key role in the chemotactic activity of neutrophils.^22^

CCL24, also known as eotaxin‐2, is a C‐C motif‐containing chemokine that facilitates eosinophil recruitment to sites of inflammatory responses to parasitic infections, as well as in allergic and autoimmune diseases, such as asthma and atopic dermatitis.^9^ Increased serum or CSF CCL24 levels have been reported in patients with fibromyalgia syndrome,^23^ neurodegenerative diseases,^24^ Huntington's disease,^25^ *Listeria monocytogenes* meningitis,^26^ neuromyelitis optica,^27^ and secondary progressive multiple sclerosis.^28^ Since eotaxin‐1 is capable of crossing the blood‐brain barrier of mice, it is plausible that eotaxins generated in the periphery may exert physiological effects in the CNS.^25^ CCL24 is a potent chemoattractant that binds to CCR3 for intracellular signaling. Various cells, including vascular endothelial cells, monocytes, and helper T cells, express CCR3 and respond to CCL24 stimulation.^23^ However, no previous reports have described changes in serum and CSF CCL24 levels in patients with neurosyphilis; we demonstrated higher CSF levels of CCL24, independently of serum levels, in these patients than in those with non‐neurosyphilis. These findings suggest that eosinophils could be recruited into the CNS and thereby play a role in the pathogenesis of neurosyphilis.^29^

CXCL7, also known as NAP‐2, is secreted from α‐granules upon platelet activation, and when it binds to its receptor, CXCR2, it plays important roles in regulating inflammation. It is a truncation product of the platelet‐derived connective tissue‐activating peptide III and is crucial in orchestrating neutrophil recruitment in response to vascular injury and neutrophil‐platelet crosstalk.^30^, ^31^ Previous studies had shown increased serum levels of CXCL7 in atherosclerosis, critical limb ischemia, and various malignancies. CXCL7 overexpression in cancer cells promotes cell proliferation in vivo and in vitro. CXCL7 binds to C‐X‐C motif chemokine receptor 1/2, inducing tumor angiogenesis and cell migration.^32^ Increased CSF CXCL7 levels were also reported in bacterial but not in viral meningitis.^33^ Lu et al found that the concentration of urokinase plasminogen activator (uPA) in the CSF of patients with neurosyphilis was significantly higher than in that of patients with non‐neurosyphilis and that uPA levels in the CSF correlated with total protein content and VDRL titer.^34^ After activation, uPA is released by neutrophils,^35^ while both CXCL7 and CXCL8 show strong neutrophil chemotactic activity. Notably, CXCL7 is identified as an endothelial cell‐released chemoattractant for human neural stem cells.^30^

Perivasculitis and endothelial cell abnormalities are characteristic histopathologic features of syphilis; *T pallidum* activates endothelial cells and increases adherence of lymphocytes and monocytes to human umbilical vein endothelial cells.^36^ Xu et al reported that exosomes derived from *T pallidum*‐infected macrophages affect adhesion to and permeability of vascular endothelial cells.^37^ This may suggest that the increased numbers of nucleated cells in the CSF of patients with neurosyphilis may be due to the continuous damage of the vascular endothelial cells of the blood‐brain barrier, rather than due to the direct chemotaxis of monocytes. Interestingly, Church et al reported that *T pallidum* directly, preferentially, and reversibly interact with activated platelets, alter their movement and blood‐brain barrier permeability, eventually facilitating their dissemination.^38^ The increased CSF levels of CCL24, CXCL7, and CXCL8 require further investigation to elucidate the role of eosinophils and neutrophils in neurosyphilis.

Our study was limited by the small sample size and retrospective design. Moreover, all samples were collected from patients in only one institution and thus may not be generalizable to the population of patients with neurosyphilis.

In conclusion, our study shows that the abnormally increased chemokine levels in the CSF of patients with neurosyphilis may disrupt blood‐brain barrier permeability, facilitating *T pallidum* dissemination, thereby playing a role in the pathogenesis of neurosyphilis.

## CONFLICTS OF INTEREST

All authors have nothing to disclose.

## AUTHORS' CONTRIBUTION

X X Li conducted the study and wrote the manuscript. X X Li and J Zhang performed the Chemokine Antibody Array; X X Li and Z Y Wang performed the ELISAs; and X X Li and S Q Chen performed the statistical analyses. W F Zhou and T T Wang collected samples from patients. M Zheng and X Y Man designed and supervised the study, and critically revised the manuscript. All patients read and approved the manuscript.

## ETHICS STATEMENT

This study was approved by the Ethics Committee of Second Affiliated Hospital of Zhejiang University School of Medicine, and written informed consent was obtained from all participants (IRB‐2016‐023).

## Supporting information

Fig S1

Supplementary Material

## Data Availability

The data that support the findings of this study are openly available in [Pan Baidu] at [https://pan.baidu.com/s/1vEuwH7wOfOzCWVgjvqzrWw], with extraction code: ha62.
