# Supplementary figures and images for: Increased CCL24 and CXCL7 levels in the cerebrospinal fluid of patients with neurosyphilis
Source: J Clin Lab Anal. 2020 May 17;34(9):e23366. doi: 10.1002/jcla.23366 (PMC7521312; doi:10.1002/jcla.23366)

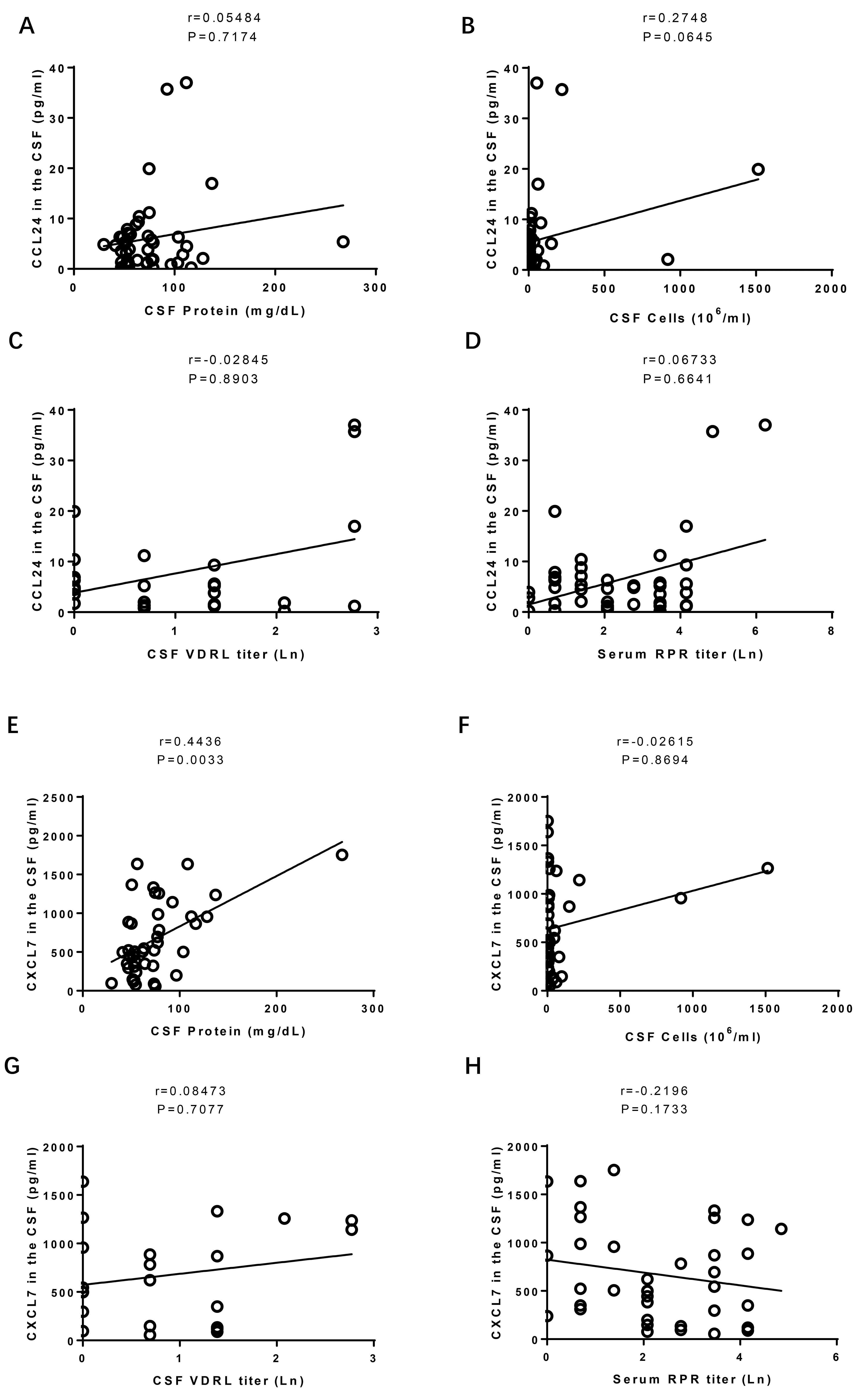

Supplement: Supplementary file 1 — Fig S1 [file JCLA-34-e23366-s001.tif]
