# Supplementary material for: Increased CCL24 and CXCL7 levels in the cerebrospinal fluid of patients with neurosyphilis
Source: J Clin Lab Anal. 2020 May 17;34(9):e23366. doi: 10.1002/jcla.23366 (PMC7521312; doi:10.1002/jcla.23366)
Supplement: Supplementary file 2 — Supplementary Material [file JCLA-34-e23366-s002.pdf]

Supplement to Increased CCL24 and CXCL7 in the cerebrospinal fluid of patients with neurosyphilis

Supplement table 1. Characteristics of the study population.

	Neurosyphilis	Non-neurosyphilis	P Value
Male (%)	32/46(70)	26/47(55)	ND
Age (Median,IQR)	57(51-64)	51(39-64)	0.1441
1/Serum RPR titer (Median,IQR)	8(2-32)	4(0-8)	<0.0002
1/CSF VDRL titer (Median,IQR)	1(0-4)	0	<0.0001
CSF cell count (Median,IQR)	8(4-41.5)	4(2-6)	<0.0001
CSF protein (Median,IQR)	68.75(52.3-82.24)	34.4(28.5-41.6)	<0.0001
CSF glu (Median,IQR)	3.69(3.08-4.05)	3.93(3.44-4.31)	0.0518
Treatment (%)	16/46(35)	22/47(47)	ND

CSF, cerebrospinal fluid; CSF protein reference range 8-43mg/dL; CSF cell count reference range <8*10^6/L; CSF glu, glucose, reference range 2.2-3.9mmol/L; IQR, interquartile range; ND, not done; RPR, rapid plasma reagin; VDRL, Venereal Disease Research Laboratories.

Supplement table 2. Demographic and clinical characteristics of the study population.

Number	Sex	Age	Confirmation time	Treatment history	Serum RPR titre	Serum TPPA	CSF RPR titre	CSF TPPA	CSF findings		
									Cell count	Protein	Glu
**1**	Female	56	3 years	Benzathine penicillin	1:512+	+	1:16+	+	54	111.7	3.35
**2**	Female	70	1 month	Benzathine penicillin	1:128+	+	1:16+	+	219	92.56	2.51
**3**	Male	63	After admission	Unknown	1:64+	+	1:16+	+	62	137.1	2.64
**4**	Male	64	3 months	Benzathine penicillin	1:64+	+	1:16+	+	40	103.2	4.02
**5**	Female	55	4 months	Unknown	1:32+	+	1:8+	+	14	78.5	2.88

**6**	Female	55	4 months	Unknown	1:32+	+	1:8+	+	14	78.5	2.88
**7**	Female	40	After admission	Unknown	1:64+	+	1:4+	+	35	52.4	2.75
**8**	Female	58	After admission	Unknown	1:64+	+	1:4+	+	60	73.6	4.05
**9**	Female	51	1 month	Unknown	1:64+	+	1:4+	+	80	64.1	5.14
**10**	Female	55	5 months	Unknown	1:32+	+	1:4+	+	4	72.9	3.54
**11**	Male	64	After admission	No prior treatment	1:32+	+	1:4+	+	150	50.2	3.88
**12**	Male	68	1 year	Unknown	1:16+	+	1:4+	+	40	53.02	3.82
**13**	Male	74	3 weeks	Unknown	1:64+	+	1:2+	+	6	47.2	3.96
**14**	Male	57	3 years	Benzathine penicillin	1:32+	+	1:2+	+	18	74.9	3.92
**15**	Male	54	9 months	Ceftriaxone	1:16+	+	1:2+	+	6	78.8	3.67
**16**	Male	52	2 months	Unknown	1:8+	+	1:2+	+	98	51.5	3.34
**17**	Male	50	1 month	Unknown	1:8+	+	1:2+	+	50	77.2	3.78
**18**	Male	36	10 months	Ceftriaxone	1:32+	+	1:1+	+	6	47.1	3.4
**19**	Male	60	7 years	Unknown	1:32+	+	1:1+	+	46	63	8.22
**20**	Female	59	11 months	Benzathine penicillin	1:16+	+	1:1+	+	4	29.7	3.29
**21**	Male	67	1 month	Unknown	1:8+	+	1:1+	+	16	103.7	2.66
**22**	Male	59	3 years	Unknown	1:8+	+	1:1+	+	4	41.2	2.5
**23**	Male	60	0.5 month	Unknown	1:4+	+	1:1+	+	8	112	4.29
**24**	Male	49	4 years	Unknown	1:4+	+	1:1+	+	10	64.9	3.43
**25**	Male	64	6 years	Unknown	1:2+	+	1:1+	+	2	56.3	3.1
**26**	Female	54	After admission	Unknown	1:2+	+	1:1+	+	1515	74.8	4.16
**27**	Male	49	3 years	Benzathine penicillin	1:32+	+	-	+	4	77.1	3.46
**28**	Female	69	10 months	Unknown	1:8+	+	-	+	12	96.6	3.05
**29**	Male	45	2 months	Benzathine penicillin	1:8+	+	-	+	4	54.6	4.02
**30**	Male	37	10 years	Benzathine penicillin	1:8+	+	-	+	4	48.6	3.61
**31**	Male	46	1 year	No prior treatment	1:8+	+	-	+	8	54	4.05
**32**	Male	58	1 month	Unknown	1:4+	+	-	+	2	267.5	3.71
**33**	Male	64	After admission	Unknown	1:4+	+	-	+	1	53.5	5.47
**34**	Female	55	3 years	Benzathine penicillin, Ceftriaxone	1:4+	+	-	+	8	61.8	3.09
**35**	Male	28	After admission	Unknown	1:4+	+	-	+	918	128.2	2.39
**36**	Male	56	1 year	Benzathine penicillin	1:2+	+	-	+	2	45.8	3.98

**37**	Male	54	1 month	Benzathine penicillin	1:2+	+	-	+	4	50.6	3.81
**38**	Female	58	10 months	Unknown	1:2+	+	-	+	10	73.8	3.19
**39**	Male	60	After admission	Unknown	1:2+	+	-	+	12	77.7	2.81
**40**	Male	39	6 months	Aqueous penicillin	1:2+	+	-	+	0	47.2	3.84
**41**	Male	38	5 months	Unknown	1:2+	+	-	+	8	53	4.53
**42**	Male	66	2 years	Benzathine penicillin	1:1+	+	-	+	4	55.1	4.12
**43**	Male	56	3 years	Benzathine penicillin	1:1+	+	-	+	6	108.2	4.45
**44**	Male	64	Several years	Unknown	1:1+	+	-	+	6	116.5	5.13
**45**	Male	65	10 years	Unknown	-	+	-	+	0	72.6	5.74
**46**	Female	57	Unknown	Unknown	-	+	-	+	6	52	2.7
**47**	Female	52	4 months	Unknown	1:128+	+	-	+	0	32.5	4.27
**48**	Female	45	17 years	Benzathine penicillin	1:16+	+	-	+	4	23.5	3.44
**49**	Male	75	5 years	Benzathine penicillin	1:8+	+	-	+	4	40	4.84
**50**	Male	40	10 months	Benzathine penicillin	1:8+	+	-	+	4	39	3.22
**51**	Male	47	1 year	Ceftriaxone	1:8+	+	-	+	6	27.8	3.78
**52**	Female	51	2 years	Benzathine penicillin	1:8+	+	-	+	6	41.6	3.35
**53**	Male	52	Unknown	Unknown	1:8+	+	-	+	6	42.4	3.2
**54**	Male	49	2 years	Unknown	1:4+	+	-	+	0	34.3	3.56
**55**	Male	48	3 years	Benzathine penicillin	1:4+	+	-	+	1	27.5	3.74
**56**	Male	55	1.5 years	Benzathine penicillin	1:4+	+	-	+	1	38.4	4.05
**57**	Male	63	1 year	Unknown	1:2+	+	-	+	6	30.5	3.08
**58**	Male	20	2 years	Benzathine penicillin	1:16+	+	-	-	2	28.5	3.78
**59**	Female	37	2 years	Unknown	1:16+	+	-	-	4	29.8	3.49
**60**	Female	67	2 years	Benzathine penicillin	1:16+	+	-	-	6	50	4.18
**61**	Male	72	1 year	Benzathine penicillin	1:8+	+	-	-	2	32.5	4.02
**62**	Female	23	3 years	Unknown	1:8+	+	-	-	2	22.7	3.3
**63**	Female	31	3 years	Benzathine penicillin	1:8+	+	-	-	0	36.2	3.49
**64**	Male	75	Over 20 years	Unknown	1:4+	+	-	-	4	36.8	4.01
**65**	Male	50	6 years	Benzathine penicillin	1:4+	+	-	-	6	27.9	3.95
**66**	Male	30	2 years	Benzathine penicillin	1:4+	+	-	-	6	38.2	3.62
**67**	Male	21	3 years	Benzathine penicillin	1:4+	+	-	-	6	39.9	3.35

**68**	Female	33	2 years	Benzathine penicillin	1:4+	+	-	-	0	28.5	3.4
**69**	Male	63	5 years	Benzathine penicillin	1:4+	+	-	-	0	55.1	4.31
**70**	Female	19	3.5 years	Unknown	1:4+	+	-	-	1	25.6	3.41
**71**	Female	72	2 years	Benzathine penicillin	1:4+	+	-	-	4	29.1	4.49
**72**	Female	47	After admission	Unknown	1:4+	+	-	-	2	18.2	4.1
**73**	Female	41	3 years	Benzathine penicillin	1:2+	+	-	-	2	32.6	3.45
**74**	Female	39	3 years	Benzathine penicillin	1:2+	+	-	-	4	32.9	4.91
**75**	Female	64	10 years	Benzathine penicillin	1:2+	+	-	-	4	62.7	3.69
**76**	Male	59	1 year	Benzathine penicillin	1:2+	+	-	-	0	35.2	4.81
**77**	Male	78	After admission	Unknown	1:1+	+	-	-	0	40.3	3.93
**78**	Female	56	10 months	Unknown	1:1+	+	-	-	4	32.9	3.8
**79**	Male	33	1 month	Unknown	1:1+	+	-	-	6	29.7	4.91
**80**	Female	31	9 years	Benzathine penicillin	1:1+	+	-	-	10	33.4	3.38
**81**	Female	66	5 years	Unknown	-	+	-	-	2	25.7	8.03
**82**	Male	78	After admission	Unknown	-	+	-	-	0	51.1	5.14
**83**	Male	56	Unknown	Unknown	-	+	-	-	2	38.8	5.28
**84**	Female	31	5 years	Unknown	-	+	-	-	4	28.2	3.08
**85**	Female	53	1 year	Unknown	-	+	-	-	2	25.3	4.1
**86**	Male	45	3 years	Unknown	-	+	-	-	4	28.4	3.95
**87**	Female	51	After admission	Unknown	-	+	-	-	2	39.4	4.53
**88**	Female	58	1 year	Unknown	-	+	-	-	2	48.7	3.55
**89**	Male	65	2 years	No prior treatment	-	+	-	-	2	71.5	5.39
**90**	Male	68	Unknown	Unknown	-	+	-	-	6	71	3.94
**91**	Male	51	20 days	Unknown	-	+	-	-	8	54.7	4.13
**92**	Male	61	After admission	Unknown	-	+	-	-	6	64.1	3.4
**93**	Male	83	After admission	Unknown	-	+	-	-	5	57.7	4.8

CSF, cerebrospinal fluid; CSF Protein reference range 8-43mg/dL; CSF cell count reference range <8*10^6/L; Glu, glucose, reference range 2.2-3.9mmol/L; RPR, rapid plasma reagin; TPPA, T pallidum particle agglutination; VDRL, venereal disease research laboratories.

Supplement table 3. Demographic and clinical characteristics of the 4 neurosyphilis and 4 non-neurosyphilis patients.

Number	Sex	Age	Confirmation time	Treatment history	Serum RPR titer	Serum TPPA	CSF VDRL titer	CSF TPPA	CSF findings		
									Cell count	Protein	Glu
**1**	Female	56	3 years	Benzathine penicillin	1:512+	+	1:16+	+	54	111.7	3.35
**2**	Female	70	1 month	Benzathine penicillin	1:128+	+	1:16+	+	219	92.56	2.51
**3**	Male	64	3 months	Benzathine penicillin	1:64+	+	1:16+	+	40	103.2	4.02
**4**	Male	63	After admission	Unknown	1:64+	+	1:16+	+	62	137.1	2.64
**5**	Female	37	2 years	Benzathine penicillin	1:16+	+	-	-	4	29.8	3.49
**6**	Female	31	3 years	Benzathine penicillin	1:8+	+	-	-	0	36.2	3.49
**7**	Male	50	6 years	Benzathine penicillin	1:4+	+	-	-	6	27.9	3.95
**8**	Male	75	More than 20 years	Unknown	1:4+	+	-	-	4	36.8	4.01

CSF, cerebrospinal fluid; CSF Protein reference range 8-43mg/dL; CSF cell count reference range <8*10^6/L; Glu, glucose, reference range 2.2-3.9mmol/L; RPR, rapid plasma reagin; TPPA, T pallidum particle agglutination; VDRL, venereal disease research laboratories.

Supplement figure 1. Correlations between the CSF CCL24, CXCL7 level and CSF protein concentration, CSF cell count, CSF VDRL titer and serum RPR titer. CSF Protein concentration, CSF cell count, CSF VDRL titer, and serum RPR titer are plotted against CSF CCL24 (A, B, C, D) and CXCL7 levels (E, F, G, H). Each circle represents one individual. The straight line in each graph is the result of linear regression analysis. Spearman correlation analysis was performed, and Spearman's correlating coefficients r and p values are shown. RPR, rapid plasma reagin; VDRL, Venereal Disease Research

Laboratories.
